# Short-term treatment of multidrug-resistant tuberculosis: a multicenter study of therapeutic effectiveness and adverse drug reactions in Yogyakarta, Indonesia

**DOI:** 10.11604/pamj.2025.52.62.45831

**Published:** 2025-10-07

**Authors:** Yunilistianingsih Yunilistianingsih, Nurul Ambariyah, Vitarani Dwi Ananda Ningrum

**Affiliations:** 1 Puskesmas (Public Health Center) Tegalrejo, Yogyakarta, Indonesia,; 2 Dr. Sardjito General Hospital, Yogyakarta, Indonesia,; 3 Department of Pharmacy, Islamic University of Indonesia, Yogyakarta, Indonesia

**Keywords:** MDR-TB, short-term regimen, ADRs, therapeutic effectiveness

## Abstract

This study analyzed the therapeutic effectiveness, ADRs, and their influencing factors in multidrug-resistant tuberculosis (MDR-TB) patients. We conducted a cross-sectional study involving patients in four referral hospitals in Yogyakarta province. The data was collected from the medical records and the Tuberculosis Information System (SITB) of the Ministry of Health of the Republic of Indonesia from January 2020 to June 2023. Of the 65 patients, 31 (47.7%) showed the expected clinical response. The factors affecting the therapeutic effectiveness were occupation and medication adherence. The most frequent ADR found was gastrointestinal disorders (19.9%). Based on the severity levels, the most frequently found ADRs were levels 1 and 3, at 60.1% and 26.1%, respectively. In conclusion, more than half of all the MDR-TB patients still showed an inadequate clinical response, with almost 40% of MDR-TB patients experiencing ADRs.

## Introduction

The estimated number of multidrug-resistant tuberculosis (MDR-TB) cases globally was 410,000 people in 2022, with a much lower number being diagnosed and starting treatment, reaching only 175,650 people. India and Indonesia are the top two countries with the greatest number of TB cases in the world. In 2023, Indonesia recorded 12,482 people suffering from MDR-TB, but only 9,134 or 73.2% of them had enrolled for the treatment [1]. However, patients who started short-term regimens in 2021 accounted for 15% of the total MDR-TB patients [2,3]. The resistance to isoniazid and rifampicin, the two first-line TB drugs, is called MDR-TB. Studies on the treatment effectiveness and adverse drug reactions (ADRs) for MDR-TB using short-term, injection-free regimens in Indonesia have been limited. In this study, we evaluate the treatment effectiveness, adverse drug reactions, and factors influencing outcomes in MDR-TB patients receiving short-term regimens.

## Methods

This research was a retrospective cross-sectional study conducted in the main referral hospitals in Yogyakarta Special Region involving all MDR-TB patients who registered from January 2020 to June 2023. The data was collected from patients' medical records and the Indonesian Tuberculosis Information System (SITB).

The therapeutic outcome was based on the 2020 Technical Guidelines for the Management of Drug-Resistant TB in Indonesia, consisting of cured, completely treated, failed, lost to follow-up, and dead.

The types of ADRs listed in the medical records and SITB were quite varied. The organ systems/functions related to ADR are:

***Cardiovascular:*** arrhythmia, cardiomegaly, QTc prolongation, tachycardia, chest pain, sinus arrhythmia, sinus tachycardia, ST-elevation, abnormal-T, agonal breathing, shortness of breath during activity, palpitations, myocardial ischemia, chest pain and shortness of breath.

***Kidney:*** increased creatinine.

***Electrolyte:*** hypokalemia, hyponatremia, hypovolemic shock, hypomagnesemia.

***Liver:*** elevated AST/ALT.

***Visual:*** stinging eyes, blurred vision and impaired vision.

***Gastrointestinal:*** nausea-vomiting, decreased appetite, gastritis, dyspepsia, abdominal pain, stress ulcer, abdominal discomfort, stomach burn, bitter mouth and bloating.

***Nervous system:*** vertigo, spinning-head, nerve pain, dizziness, sleep disorder, tingling numbness in the soles, suicidal thoughts, depression, pins and needles, numbness in the feet, motor aphasia, speech disorder, anxiety, neuropathy, migraine, adjustment disorder and headache.

***Endocrine:*** hypothyroid.

***Skin:*** itching, hyperpigmentation, back-acne, mild allergic skin reaction, itching and red spots and dry scaly skin.

***Muscle and bone:*** joint pain, knee pain, arm pain, waist pain, hyperuricemia, wrist pain, shoulder pain, joint pain and swelling, body aches, stiff hands/neck, sore muscles, bilateral osteoarthritis, myalgia, arthralgia and tingling legs.

***Hematologic:*** anaemia, leukocytosis, thrombocytosis and febrile neutropenia.

***Auditory tract:*** ringing in the ears.

Meanwhile, the ADRs severity was divided into level-1 (mild): mild or temporary discomfort (>48 hours), requiring no medical intervention or treatment, level-2 (moderate): limited activity (mild-moderate), requiring further examination, and requiring no medical intervention or treatment, level-3 (severe): limited activity, requiring medical intervention or mild treatment, and possibly requiring hospitalization, and level-4 (potentially life-threatening): extremely limited activity, strongly requiring assistance, significantly requiring medical intervention or treatment, and most likely requiring hospitalization.

The data analysis used SPSS version 25.0 through a multivariate analysis with a logistic regression test to analyse the factors that affected both the treatment effectiveness and the incidence of ADRs in MDR-TB patients.

Ethical approval was obtained from all research sites.

## Results

**Therapeutic effectiveness:** data were collected in three secondary hospitals and one tertiary hospital. Of 65 subjects, therapeutic effectiveness was only found in 31 patients (47.7%). The ineffective MDR-TB therapy in 34 patients consisted of 29 patients who were declared to have failed treatment, 3 patients who had lost to follow-up, and 2 patients who died. The Chi-square test showed a correlation between patient characteristics and the effectiveness of therapy (p<0.05, Table 1). The correlated patient characteristics were medication adherence (p 0.028; OR 9.231; 95% CI 1.082 - 78.784) and occupation (p 0.028; OR 4.343, 95% CI 1.474-12.793). Considering the value of OR (EXP)^B^, the greater correlation strength is the occupation (OR = 0.160). Patients who adhered to the treatment were 9.23 times more likely to achieve therapeutic effectiveness than those who did not comply. In addition, formal workers were 4.34 times more likely to experience ineffectiveness compared to informal workers.

**Table 1:** association of the patients' demographic and clinical characteristics with the therapeutic effectiveness recruited from four hospitals in Yogyakarta-Indonesia, January 2020-June 2023 (N=65)

Characteristic	Category	n	Therapeutic response effectiveness		p value	OR	95% CI	
			Effective	Ineffective			Lower	Upper
Age	< 60 years	57	26	31	0.463	1.987	0.433	9.116
	≥ 60 years	8	5	3				
Gender	Female	35	15	20	0.553	0.656	0.246	1.751
	Male	30	16	14				
Educational Attainment	Low	14	8	6	0.619	0.616	0.187	2.032
	High	51	23	28				
Occupation	Formal	26	7	19	0.013*	4.343	1.474	12.793
	Informal	39	24	15				
Distance from home to hospital	≥ 10 km	38	19	19	0.849	1.250	0.464	3.365
	< 10 km	27	12	15				
Distance from home to primary healthcare facility	≥ 5 km	14	6	8	0.915	0.780	0.237	2.570
	< 5 km	51	25	26				
History of TB treatment	Previous treatment	29	16	13	0.404	1.723	0.642	4.624
	New TB	36	15	21				
DM comorbidity	Yes	17	7	10	0.731	1.429	0.466	4.376
	No	48	24	24				
Regimen category	STR by guidelines	60	29	31	1.000	0.713	0.111	4.575
	IndividualizedSTR	5	2	3				
BMI	< 18.50	30	18	12	0.112	0.394	0.145	1.073
	>18.50	35	13	22				
Medication adherence	No	9	1	8	0.028*	9.231	1.082	78.784
	Yes	56	30	26				

* P value < 0.05

**Adverse Drug Reactions (ADRs):** in this study, ADRs were experienced by all the patients. Among them, 276 ADRs were found. The data obtained showed that 41 patients (58.6%) had ADRs in less than six organs, and 24 patients (34.3%) had ADRs in six or more organs. The ADRs recorded belonged to level-1, followed by level-3 and 2. Logistic regression analysis indicated that distance to the hospital and medication adherence were correlated with levels 2 and 3 of ADR (p<0.05, Table 2). Even though both variables have a p-value of less than 0.05, the odds ratio (OR) for each variable carries different meanings. Adhering to prescribed medication can reduce the risk of adverse drug reactions (ADRs) by as much as 7.8 times.

**Table 2:** logistic regression analysis of the ADR incidence of level 2 and 3 on mdr-tb short-term regimen use recruited from four hospitals in Yogyakarta-Indonesia, January 2020-June 2023 (N=65)

Characteristic	Coefficient	S.E	Wald	df	P value	OR	95% CI	
							Min	Max
Distance from home to hospital	2.023	0.926	4.777	1.000	0.029	7.563	1.232	46.410
Medication adherence	-2.056	0.950	4.687	1.000	0.030	0.128	0.020	0.823
Constant	-21.633	15955.213	0.000	1.000	0.999	0.000		

## Discussion

Several studies related to the effectiveness of short-term MDR-TB therapy show various results. These findings are different from a study conducted in the Kyrgyz Republic, where MDR-TB patients treated with a short-term regimen (4-6 (Bdq-Mfx-Cfz-EH-(HD)-Pto)/5 (Mfx-Cfz-E-Pto)) showed 75% therapeutic effectiveness [4]. A study in South Africa with the same regimen (9-12 months without injections) gave 74% effectiveness [5]. In addition, this result showed that working patients will be busier with their work, making them forget the follow-up schedule and forget to take their medication, thus reducing the success rate. As expected, the compliant patients in this study were 9.23 times more likely to experience therapeutic effectiveness than non-compliant patients. This finding is in accordance with the research, which shows that patient compliance plays an important role in the success of TB treatment [6,7].

On the other hand, the ADRs found in this study follow the findings of previous studies conducted on MDR-TB patients with short-term treatment combinations. A study of the therapeutic outcomes and side effects among MDR-TB patients in Nigeria with short-term treatment regimens showed that the side effects experienced by the patients included vomiting, hearing loss, and liver disorders [8]. Similarly, the side effects experienced by the MDR-TB patients undergoing short-term treatment regimens in Uzbekistan were nausea and vomiting, fatigue, abdominal pain, headache, arthralgia, kidney disease, anorexia, hearing loss, diarrhea, rash, anemia, QTc prolongation, anemia, depression/anxiety, visual impairment, cramps, gastritis, mental disorders, hypothyroidism, allergic reactions, seizures, and bleeding [9].

In addition, the incidence of ADR in the prospective cohort study was not different from this study, namely gastrointestinal disorders, nervous system disorders, and electrolyte imbalances being the highest types of ADR in patients with MDR-TB [10]. Meanwhile, the ADR grouping in this study is almost the same as those shown in studies in African countries, including Nigeria, with the most ADRs being level-1 followed by level-2, level-3, and level-4 [11,12]. The difference related to this study was not found to be ADR level-4 and the incidence of level-3 was higher when compared to level-2. The high incidence of ADR in this study requires serious attention, although not all of it is related to treatment adherence, as it can lead to a decrease in the quality of life of patients, even until the end of treatment. In general, ADR level-3 is a severe category, which has the most potential to cause patients to be reluctant to continue treatment. Further research could prospectively confirm this assumption.

Based on the statistical tests, there was no correlation between demographic and clinical factors of the patients and the incidence of ADRs in MDR-TB patients. However, these findings differ from a 2018 study in Pakistan, which revealed a correlation between age, delayed reporting to TB centers, and the occurrence of ADR [13]. Similarly, retrospective research in Italian tertiary hospitals with 8 years of data involving 74 patients showed that homelessness affected the incidence of ADR [14].

Furthermore, this study revealed that patients living 10 km or more from the hospital are at a 7,563 times higher risk of experiencing level-2 or level-3 ADR. Longer distances result in delays and limited patient access to healthcare providers. Additionally, a study in Uganda indicated that the lack of access to health facilities and direct monitoring of treatment by healthcare providers was significantly linked to the incidence of ADR in MDR-TB treatment [15].

In addition to the limited number of subjects involved, there was no control group and ADR causality instruments made the incidence of ADR unstated due to the use of drug regimen or other causes such as disease prognosis. Studies with larger sample sizes and diverse genetic profiles can complement and reveal disparities in ADR incidence. Furthermore, the positive aspect of this finding is that adherence to treatment exhibits a protective effect against ADR events (OR 0.128). Apart from enhancing the effectiveness of therapy, medication adherence can reduce the risk of ADR by up to 7.8 times.

## Conclusion

In conclusion, more than half of all the MDR-TB patients in Yogyakarta still showed an inadequate clinical response, with ADR experienced by almost forty per cent. Treatment adherence has a positive impact both in terms of treatment effectiveness and protection against ADR. Therefore, immediate remedial steps focusing on medication adherence can be taken to improve the success of therapy with minimal ADRs.

## What is known about this topic

- The use of all-oral drugs, no longer injectable, has been announced by the WHO since 2018;
- More than 50% of patients with MDR-TB treatment experience ADR of varying severity.

## What this study adds

- Although there has been an Indonesian government policy regarding the provision of MDR-TB medication services accompanied by health workers at health facilities, most of the MDR-TB patients in Yogyakarta still do not adhere to medication;
- As a province in Indonesia, Yogyakarta still has a relatively low success rate of MDR-TB therapy;
- Adherence to MDR-TB treatment can increase the cure rate by up to 9.2 times and offers a protective effect against adverse drug reactions (ADRs) by 7.8 times.
